# Earlier screening, better outcomes? Revisiting breast cancer screening guidelines for women in their 40s

**DOI:** 10.17269/s41997-025-01112-7

**Published:** 2025-10-20

**Authors:** Nguyet N. M. Ngo, Jennifer D. Brooks

**Affiliations:** Dalla Lana School of Public Health, University of Toronto, Toronto, ON Canada

**Keywords:** Breast cancer, Mammography screening, Early detection, Young breast cancer, Health policy, Cancer du sein, Dépistage par mammographie, Détection précoce, Cancer du sein chez les jeunes, Politique de santé

## Abstract

While mammography screening programmes improve early detection and reduce mortality for individuals aged 50–74, its extension to those aged 40–49 remains debated. In Canada, breast screening eligibility varies between provinces/territories, with Ontario lowering its eligibility age from 50 to 40 in 2024. This commentary examines recent evidence, including observational studies and simulation models, suggesting that mammography screening from age 40 may offer net benefits. Additionally, using data from Ontario Health (Cancer Care Ontario), we compared stage at diagnosis and 5-year survival rates among 18,639 women aged 40–51 diagnosed with breast cancer (2009–2017). Individuals aged 40–49 had comparable stage at diagnosis and 5-year survival rates to unscreened individuals aged 50–51. Meanwhile, screened individuals aged 50–51 demonstrated the earliest stage at diagnosis and highest 5-year survival rate. Our analysis illustrates the arbitrary nature of an age-based screening threshold at 50. We demonstrate that outcomes for women aged 40–49 resemble those of unscreened women aged 50–51, who were just above the eligibility cutoff. While expanding screening may increase upfront costs, these could be offset by avoiding late-stage treatments and integrating risk-stratified approaches. Overall, women in their 40s may benefit from organized screening programs through earlier detection and improved survival.

## Résumé

Bien que les programmes de dépistage par mammographie améliorent la détection précoce et réduisent la mortalité chez les personnes âgées de 50 à 74 ans, leur extension aux femmes de 40 à 49 ans fait encore l’objet de débats. Au Canada, l’admissibilité au dépistage du cancer du sein varie selon les provinces et territoires, et l’Ontario a abaissé l’âge d’admissibilité de 50 à 40 ans en 2024. Ce commentaire examine des données récentes, incluant des études observationnelles et des modèles de simulation, suggérant que le dépistage dès 40 ans pourrait offrir des bénéfices nets. De plus, à partir des données d’Ontario Santé (Action Cancer Ontario), nous avons comparé le stade au diagnostic et les taux de survie à cinq ans chez 18 639 femmes âgées de 40 à 51 ans ayant reçu un diagnostic de cancer du sein entre 2009 et 2017. Les femmes de 40 à 49 ans présentaient des stades de diagnostic et des taux de survie similaires à ceux des femmes de 50 à 51 ans non dépistées. En revanche, les femmes de 50 à 51 ans dépistées présentaient les stades les plus précoces au diagnostic et les meilleurs taux de survie à cinq ans. Notre analyse illustre le caractère arbitraire du seuil d’âge de 50 ans pour l’admissibilité au dépistage. Nous avons montré que les résultats des femmes de 40 à 49 ans ressemblent à ceux des femmes non dépistées de 50 à 51 ans, juste au-dessus du seuil. Bien que l’élargissement du dépistage puisse entraîner des coûts initiaux plus élevés, ceux-ci pourraient être compensés par une réduction des traitements à un stade avancé et l’intégration d’approches fondées sur le risque. Globalement, les femmes dans la quarantaine pourraient bénéficier de programmes de dépistage organisés grâce à une détection plus précoce et une survie améliorée.

## Commentary

Breast cancer is a significant health concern in Canada, projected to account for 25.4% of incident cancer cases and 13.5% of cancer-specific mortality among women in 2024 (Brenner et al., 2024). In Canada, breast cancer screening programmes are available in all provinces and most territories for women aged 50–74. Depending on the province/territory, women aged 40–49 can self-refer or require a referral from their healthcare provider to receive screening. While women aged 40–49 are eligible for mammography screening in some jurisdictions, this age group is still recommended to make individual screening decisions with their clinician about when to start screening based on their health history. As such, the question of screening initiation age and frequency for women aged 40–49 remains controversial.

The 2024 Canadian Task Force on Preventive Healthcare’s draft recommendation maintained that there is no sufficient evidence to support routine screening for average-risk women in their 40 s and advised this age group to discuss with their healthcare providers the potential risks and benefits of screening before initiating screening (Canadian Task Force on Preventive Health Care, 2024). In contrast, in 2024, the U.S. Preventive Services Task Force (USPSTF) updated its guidelines to support biennial mammography screening from age 40, citing collaborative modeling data that estimated 1.3 additional breast cancer deaths averted per 1000 women screened when biennial screening starts at age 40, rather than age 50 (US Preventive Services Task Force, 2024). The USPSTF also highlighted a 2.0% annual mean increase in the incidence rate of invasive breast cancer among women aged 40–49 in recent years, concluding that screening starting at age 40 would have moderate net benefit in reducing breast cancer mortality (US Preventive Services Task Force, 2024). Breast cancer rates are also increasing in Canadians under 50 (Seely et al., 2024), making the issue of age at screening initiation an important one.

## Screening for women aged 40–49 may improve survival and stage at diagnosis

While there are valid concerns regarding routine screening in women aged 40–49, such as resource allocation and radiological challenges (i.e. high breast density in this age group, making mammograms harder to read) (Grimm et al., 2022), compelling evidence suggests a net benefit of extending organized screening to this age group. Wilkinson et al. (2023a, b) compared net survival rates in women aged 40–49 diagnosed with breast cancer, living in Canadian jurisdictions with organized breast screening programmes beginning at age 40 versus those starting at age 50. They reported a significant survival advantage among the former, particularly among women aged 45–49 (2.6 percentage points (pp) increase) who lived in jurisdictions with screening programmes that include women in their 40s. Additionally, they found no difference in cancer incidence between the two groups, suggesting improved outcomes with minimal risk of overdiagnosis. This study reinforces the value of including women aged 40–49 in organized screening programmes in improving 10-year net survival (Wilkinson et al., 2023a, b).

Similarly, simulation modeling using the OncoSim-Breast model on a cohort of 1.53 million women demonstrated that biennial screening from age 40 to 74 could reduce breast cancer mortality by 5.9 fewer deaths per 1000 individuals compared to no screening, and by 1.4 fewer deaths per 1000 individuals compared to screening from age 50 to 74 (Yaffe & Mainprize, 2023). Their findings also demonstrated that diagnosis of advanced-stage cancers decreased when screening initiated at age 40 compared to 50.

Extending on this simulation model, we used real-world data from Ontario Health (Cancer Care Ontario) to conduct a retrospective cohort of 18,639 women aged 40–51 diagnosed with a first primary breast cancer between 2009 and 2017 and followed up until December 31, 2019. We excluded individuals in the High Risk Ontario Breast Screening Program (OBSP), that is, women with known elevated risk due to genetic mutations (e.g. *BRCA1/2*) or strong family history, as they follow a different screening guideline. Including high-risk individuals would introduce heterogeneity that makes it difficult to isolate the effects of routine screening among average-risk women.

With the cohort defined, we categorized participants aged 40–51 into 2-year age groups based on their age at breast cancer diagnosis (e.g. 40–41, 42–43). The 50–51-year-old group was further divided into those who had been screened as part of the OBSP (i.e. screeners) and those who had not (i.e. non-screeners). We then used descriptive statistics to look at how stage at diagnosis and breast cancer-specific mortality compared between these groups. The proportion of stage IV cancers in each age group was compared using the pair-wise proportions test and survival compared using the log-rank test, both adjusting for multiple comparisons using Holm’s method.

**Fig. 1:**
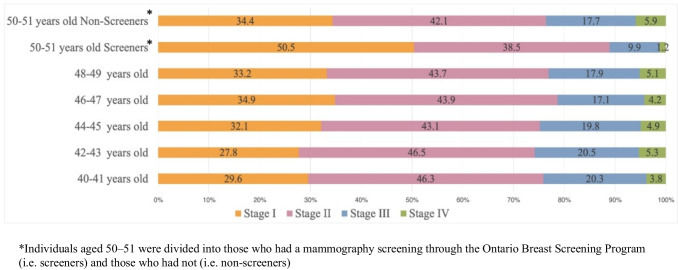
Stage at diagnosis for individuals aged 40–51 years diagnosed with breast cancer in Ontario, Canada between 2009 and 2017 and followed up until December 31, 2019

Table 1 shows the distribution of stage at diagnosis and breast cancer specific mortality by age group. We found that individuals aged 40–49 and non-screeners aged 50–51 were primarily diagnosed with Stage II (Fig. 1). In contrast, most women aged 50–51 who participated in screening were diagnosed at stage I disease (50.5%). Corresponding to this, women aged 40–49 and non-screeners aged 50–51 had comparable rates (3.8–5.9%) of stage IV diagnoses, while screeners aged 50–51 had the lowest proportion of stage IV diagnoses (1.2%) (*P* < 0.05).

**Table 1:** Stage and mortality rate of participants aged 40–51 years diagnosed with breast cancer between 2009 and 2017 and followed up until December 31, 2019

Age group	40–41 (***N*** = 1592)	42–43 (***N*** = 2109)	44–45 (***N*** = 2709)	46–47 (***N*** = 3309)	48–49 (***N*** = 3823)	50–51 Screeners^1 ^(***N*** = 2066)	50–51 Non-screeners^1 ^(***N*** = 3031)
Stage at diagnosis							
I	369 (29.6%)	455 (27.8%)	679 (32.1%)	902 (34.9%)	999 (33.2%)	790 (50.5%)	792 (34.4%)
II	576 (46.3%)	761 (46.5%)	913 (43.1%)	1136 (43.9%)	1316 (43.7%)	602 (38.5%)	969 (42.1%)
III	253 (20.3%)	335 (20.5%)	420 (19.8%)	442 (17.1%)	540 (17.9%)	155 (9.9%)	407 (17.7%)
IV	47 (3.8%)^2^	86 (5.3%)^2^	104 (4.9%)^2^	108 (4.2%)^2^	155 (5.1%)^2^	18 (1.2%)	136 (5.9%)^2^
Unknown	347	472	593	721	813	501	727
Breast cancer mortality	124 (7.8%)	157 (7.4%)	229 (8.5%)	199 (6%)	261 (6.8%)	50 (2.4%)	255 (8.4%)

^1^Individuals aged 50–51 were divided into those who had a mammography screening through the Ontario Breast Screening Program (i.e. screeners) and those who had not (i.e. non-screeners)

^2^Statistically significant difference (*P* < 0.05) when compared to ‘Screeners aged 50–51’ using pairwise proportions test and Holm’s correction for multiple comparisons

**Fig. 2:**
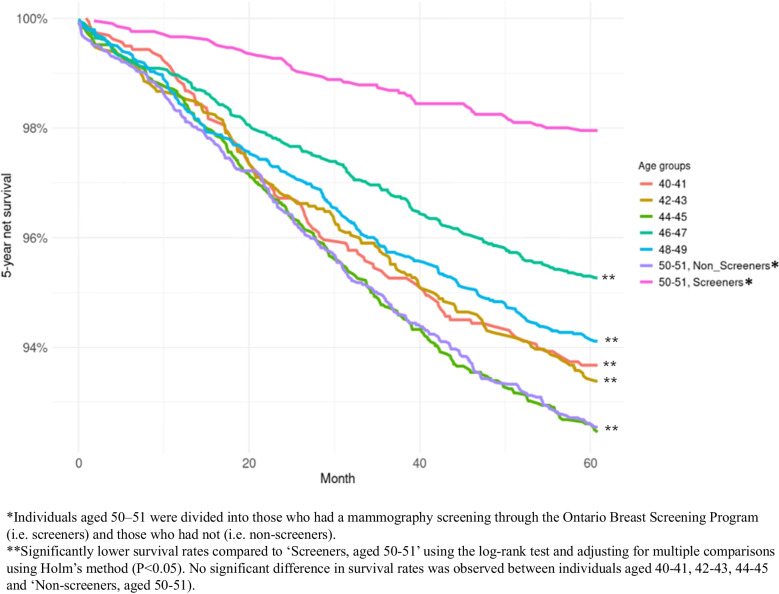
Five-year net survival rates among women aged 40–51 years diagnosed with breast cancer between 2009 and 2017 in Ontario, Canada, and followed up until December 31, 2019

When we looked at survival outcomes, screened individuals aged 50–51 had the lowest breast cancer-specific mortality (2.4%) (*P* < 0.05), while women aged 44–45 and non-screeners aged 50–51 showed the highest mortality rates (8.5% and 8.4%, respectively) (Fig. 2). Although 5-year survival rates exceeded 92% across all age groups, screened women aged 50–51 had the highest survival rate by far at almost 98%. Notably, no significant difference in survival rates was observed for individuals aged 40–41, 42–43, and 44–45 when compared to non-screeners aged 50–51. For individuals aged 46–47 and 48–49 years, survival rates were significantly higher than non-screeners, but still significantly lower than screeners (*P* < 0.05) (Fig. 2). It is thought that better outcomes in these older 40 s age groups are likely due to opportunistic screening occurring outside of the OBSP; however, we did not have the data to look at this in the current analysis. Overall, these findings demonstrate that the outcomes for women in their 40 s resemble those of non-screeners aged 50–51. Given that screeners aged 50–51 demonstrated the best 5-year survival rate and stage at diagnosis, extending screening to women aged 40–49 may yield comparable benefits of earlier detection and treatment.

## Challenges in expanding organized breast screening programmes for women aged 40–49

Expanding organized screening to women in their 40 s inevitably raises concerns about healthcare costs and capacity of the public health system. Ontario estimated that additional 130,000 mammograms will be conducted annually as a result of lowering the OBSP eligibility age to 40 (Government of Canada, 2023). However, increased screening costs may be offset by reductions in treatment costs associated with later-stage diagnoses. Recent cost analyses indicate that treating stage IV breast cancer is 7.7–10.9 times more expensive than treating stage I, while stage III treatment costs 1.5–4.2 times more than stage I (Wilkinson et al., 2023a, b). Additionally, an economic analysis using the Onco-Sim Breast microsimulation model suggested that biennial screening from ages 40–74 is a cost-saving public health intervention, with CAD$49,759 saved per death averted, $1558 per life-year saved, and $2007 per quality-adjusted life-year (QALY) gained (Wilkinson et al., 2025). These findings suggest that initiating screening earlier not only improves survival but also reduces overall healthcare spending by lowering costs associated with later-stage breast cancer treatment. Moving away from a strictly age-based approach to risk-based screening is another potential strategy to help manage the additional costs of expanded screening and manage the potential benefit/harm ratio of screening. A risk-based screening approach tailors the screening starting age, frequency, and modality (e.g. mammogram, MRI) based on multiple risk factors (e.g. family history, breast density, lifestyle factors) rather than just age. This approach can identify women at higher risk who are more likely to benefit from earlier screening, while avoiding unnecessary screening for those at lower risk (Mühlberger et al., 2021). In practice, this means that although all women in Ontario are eligible for mammography screening in the OBSP starting at age 40, by using a risk-based approach, individuals could make an informed decision with their healthcare provider about when to start screening based on their individual risk. There are multiple ongoing studies examining the impact of a risk-based approach to screening both in Canada (Walker et al., 2024) and internationally (Esserman et al., 2021; French et al., 2020).

## Conclusion: The need to re-evaluate breast screening guidelines for women aged 40–49

Overall, the inconsistency in Canadian breast cancer screening guidelines across provinces/territories reflects the ongoing challenge of optimizing screening benefits while minimizing harms. However, our analysis using real-world data from Ontario Health (Cancer Care Ontario) demonstrates the arbitrary nature of an age-based approach to determining screening eligibility, finding that women with breast cancer aged 40–49 have comparable stage at diagnosis and mortality rates to unscreened women aged 50–51, while screened women aged 50–51 demonstrated better cancer outcomes. This suggests that women in their 40 s could benefit from the earlier detection and treatment that come with participation in an organized screening programme.

## Contribution to knowledge

This commentary offers timely evidence to the breast cancer screening literature in Canada by highlighting that the current eligibility age of 50 for organized screening programmes may lead to missed opportunities for earlier detection and improved survivorship. Using retrospective cohort data from Ontario Health, we demonstrated that women in their 40 s diagnosed with breast cancer had similar stage at diagnosis and 5-year mortality rates as unscreened women aged 50–51, while screened women aged 50–51 experienced the most favourable outcomes. These findings, when considered alongside recent analyses showing that biennial screening from ages 40–74 is cost-effective to the Canadian healthcare system, underscore the potential benefit of extending organized screening to women in their 40s. By presenting real-world data in support of earlier detection and improved outcomes, this commentary contributes important evidence to inform Canada’s breast cancer screening guidelines.

## Data Availability

The data that support the findings of this study are available from Ontario Health (Cancer Care Ontario), a prescribed entity under Sect. 45 of the Personal Health Information Protection Act. Ontario Health (Cancer Care Ontario) is prohibited from making the data used in this research publicly accessible if it includes potentially identifiable personal health information and/or personal information as defined in Ontario law, specifically the Personal Health Information Protection Act and the Freedom of Information and Protection of Privacy Act. Upon request, data deidentified to a level suitable for public release may be provided.

## Code availability

Available upon request.

## References

[CR1] Canadian Task Force on Preventive Health Care. (2024). *Breast cancer (update) – draft recommendations (2024). *https://canadiantaskforce.ca/guidelines/published-guidelines/breast-cancer-update-2024/. Accessed 9 May 2025.

[CR2] Esserman, L., Eklund, M., Veer, L. V., Shieh, Y., Tice, J., Ziv, E., Blanco, A., Kaplan, C., Hiatt, R., Fiscalini, A. S., Yau, C., Scheuner, M., Naeim, A., Wenger, N., Lee, V., Heditsian, D., Brain, S., Parker, B. A., LaCroix, A. Z., … Plaza, M. (2021). The WISDOM study: A new approach to screening can and should be tested. *Breast Cancer Research and Treatment,**189*(3), 593–598. 10.1007/s10549-021-06346-w 34529196 10.1007/s10549-021-06346-w

[CR3] French, D. P., Astley, S., Brentnall, A. R., Cuzick, J., Dobrashian, R., Duffy, S. W., Gorman, L. S., Harkness, E. F., Harrison, F., Harvie, M., Howell, A., Jerrison, A., Machin, M., Maxwell, A. J., McWilliams, L., Payne, K., Qureshi, N., Ruane, H., Sampson, S., … Evans, D. G. (2020). What are the benefits and harms of risk stratified screening as part of the NHS breast screening programme? Study protocol for a multi-site non-randomised comparison of BC-predict versus usual screening (NCT04359420). *Bmc Cancer,**20*(1), Article 570. 10.1186/s12885-020-07054-2 32552763 10.1186/s12885-020-07054-2 PMC7302349

[CR4] Government of Canada, S. C. (2023). *Five-year cancer survival by stage at diagnosis in Canada. *https://www150.statcan.gc.ca/n1/pub/82-003-x/2023001/article/00001-eng.htm. Accessed 9 May 2025.

[CR5] Grimm, L. J., Avery, C. S., Hendrick, E., & Baker, J. A. (2022). Benefits and risks of mammography screening in women ages 40 to 49 years. *Journal of Primary Care & Community Health,**13*, 21501327211058320. 10.1177/21501327211058322 10.1177/21501327211058322 PMC8796062 35068237

[CR6] Mühlberger, N., Sroczynski, G., Gogollari, A., Jahn, B., Pashayan, N., Steyerberg, E., Widschwendter, M., & Siebert, U. (2021). Cost effectiveness of breast cancer screening and prevention: A systematic review with a focus on risk-adapted strategies. *The European Journal of Health Economics: HEPAC: Health Economics in Prevention and Care,**22*(8), 1311–1344. 10.1007/s10198-021-01338-5 34342797 10.1007/s10198-021-01338-5

[CR7] Seely, J. M., Ellison, L. F., Billette, J.-M., Zhang, S. X., & Wilkinson, A. N. (2024). Incidence of breast cancer in younger women: A Canadian trend analysis. *Canadian Association of Radiologists Journal*. 10.1177/08465371241246422 38664982 10.1177/08465371241246422

[CR8] US Preventive Services Task Force. (2024). Screening for breast cancer: US Preventive Services Task Force recommendation statement. *Jama,**331*(22), 1918–1930. 10.1001/jama.2024.5534 38687503 10.1001/jama.2024.5534

[CR9] Walker, M. J., Blackmore, K. M., Chang, A., Lambert-Côté, L., Turgeon, A., Antoniou, A. C., Bell, K. A., Broeders, M. J. M., Brooks, J. D., Carver, T., Chiquette, J., Després, P., Easton, D. F., Eisen, A., Eloy, L., Evans, D. G., Fienberg, S., Joly, Y., Kim, R. H., … Chiarelli, A. M. (2024). Implementing multifactorial risk assessment with polygenic risk scores for personalized breast cancer screening in the population setting: Challenges and opportunities. *Cancers,**16*(11), Article 2116. 10.3390/cancers16112116 38893236 10.3390/cancers16112116 PMC11171515

[CR10] Wilkinson, A. N., Ellison, L. F., Billette, J.-M., & Seely, J. M. (2023a). Impact of breast cancer screening on 10-year net survival in Canadian women age 40–49 years. *Journal of Clinical Oncology,**41*(29), 4669–4677. 10.1200/JCO.23.00348 37540825 10.1200/JCO.23.00348 PMC10564321

[CR11] Wilkinson, A. N., Seely, J. M., Rushton, M., Williams, P., Cordeiro, E., Allard-Coutu, A., Look Hong, N. J., Moideen, N., Robinson, J., Renaud, J., Mainprize, J. G., & Yaffe, M. J. (2023b). Capturing the true cost of breast cancer treatment: Molecular subtype and stage-specific per-case activity-based costing. *Current Oncology (Toronto, Ont.),**30*(9), 7860–7873. 10.3390/curroncol30090571 37754486 10.3390/curroncol30090571 PMC10527628

[CR12] Wilkinson, A. N., Mainprize, J. G., Yaffe, M. J., Robinson, J., Cordeiro, E., Look Hong, N. J., Williams, P., Moideen, N., Renaud, J., Seely, J. M., & Rushton, M. (2025). Cost-effectiveness of breast cancer screening using digital mammography in Canada. *JAMA Network Open,**8*(1), Article e2452821. 10.1001/jamanetworkopen.2024.52821 39745700 10.1001/jamanetworkopen.2024.52821 PMC11696453

[CR13] Yaffe, M. J., & Mainprize, J. G. (2023). Effect of breast screening regimen on breast cancer outcomes: A modeling study. *Current Oncology,**30*(11), 9475–9483. 10.3390/curroncol30110686 37999106 10.3390/curroncol30110686 PMC10670884

